# Performance of abbreviated protocols versus unenhanced MRI in detecting occult breast lesions of mammography in patients with dense breasts

**DOI:** 10.1038/s41598-022-17945-y

**Published:** 2022-08-11

**Authors:** Peipei Chen, Guangrui Shao, Baijie Li, Huikun Zhang, Juan Xiao, Suhong Zhao

**Affiliations:** 1 Department of Radiology, The Second Hospital, Cheeloo College of Medicine, Shandong University, No. 247, Beiyuan Road, Tianqiao District, Jinan, 250033 Shandong China; 2 Center of Evidence-Based Medicine, Institute of Medical Sciences, The Second Hospital, Cheeloo College of Medicine, Shandong University, No. 247, Beiyuan Road, Tianqiao District, Jinan, 250033 Shandong China

**Keywords:** Diagnosis, Medical imaging, Breast cancer, Cancer screening

## Abstract

To assess the diagnostic ability of abbreviated protocols of MRI (AP-MRI) compared with unenhanced MRI (UE-MRI) in mammographically occult cancers in patients with dense breast tissue. The retrospective analysis consisted of 102 patients without positive findings on mammography who received preoperative MRI full diagnostic protocols (FDP) between January 2015 and December 2018. Two breast radiologists read the UE, AP, and FDP. The interpretation times were recorded. The comparisons of the sensitivity, specificity and area under the curve of each MRI protocol, and the sensitivity of these protocols in each subgroup of different size tumors used the Chi-square test. The paired sample *t*-test was used for evaluating the difference of reading time of the three protocols. Among 102 women, there were 68 cancers and two benign lesions in 64 patients and 38 patients had benign or negative findings. Both readers found the sensitivity and specificity of AP and UE-MRI were similar (*p* > 0.05), whereas compared with FDP, UE had lower sensitivity (Reader 1/Reader 2: *p* = 0.023, 0.004). For different lesion size groups, one of the readers found that AP and FDP had higher sensitivities than UE-MRI for detecting the lesions ≤ 10 mm in diameter (*p* = 0.041, *p* = 0.023). Compared with FDP, the average reading time of UE-MRI and AP was remarkably reduced (*p* < 0.001). AP-MRI had more advantages than UE-MRI to detect mammographically occult cancers, especially for breast tumors ≤ 10 mm in diameter.

## Introduction

Mammography remains the primary screening tool for breast cancer, and it has resulted in a decline in the mortality of breast cancer^1^. However, for women with dense breasts, cancers are more likely to be obscured by the overlying tissues^2,3^. Independent of other risk factors, for women with density breast tissue, the mortality rate from breast cancer is nearly twice as high as women with low density breasts^4^.

Supplemental screening with ultrasound after mammography in women with dense breasts can improve the rate of detection of breast cancer, although this benefit may be offset by a high false positive rate^5,6^. MRI is more effective for detecting breast cancer than mammography or ultrasound^7^. According to the American College of Radiology guidelines, supplemental MRI does not take breast density into account, but is recommended annually for patients at high risk for breast cancer^8^. Therefore, it is necessary to develop a cost-effective and reasonable screening technique to detect mammographically occult malignant tumor in women with dense breasts.

After the abbreviated protocol (AP)-MRI (consisting of first post-contrast subtracted [FAST] and maximum-intensity projection [MIP] images) was first proposed by Kuhl et al.^9^, several studies demonstrated that AP had high sensitivity in breast carcinomas detection and was not affected by breast density^10–12^. However, unenhanced magnetic resonance imaging (UE-MRI) grounded on diffusion-weighted imaging (DWI), has also been widely studied for screening women with dense breasts^13–15^. The purpose of this study was to evaluate the ability of the two examinations to detect mammographically occult cancers in patients with dense breasts in comparison with FDP-MRI.

## Results

There were 102 patients including 64 patients with mammographically occult cancer and 38 benign or negative patients. The median age of the cancer cohort was 48.2 years (range: 30–73 years). There were 24 women with extremely dense breasts and 78 with heterogeneously dense breasts included in this study. Two patients were found to have bilateral cancers and two patients have two ipsilateral breast cancers. Ten patients carried high risk BRCA1 mutant genes, and 17 women had a history of family or personal breast cancer, and they all presented with benign or negative mammograms.

### Pathological findings

There were 108 lesions in 102 women; 82 lesions underwent surgical resection and 26 underwent ultrasound-guided biopsy. The histopathological diagnoses were 68 cancers and 40 benign lesions (Table 1). Among the invasive ductal carcinoma (IDC) patients, two of them had ipsilateral breast fibroadenoma, and eight had ipsilateral or contralateral ductal carcinoma in situ (DCIS). Among the pathological benign lesions, there are six women had no positive findings on FDP-MRI and mammography; however, they underwent ultrasound-guided biopsy as a result of the suspicious lesions found by ultrasound examination. The six lesions included 3 atypical ductal hyperplasia, 2 fibroadenomatous hyperplasia and 1 ductal dilatation.

**Table 1:** Histopathological diagnosis of all breast lesions.

Histopathological subtype	n (%)
Benign	40
Fibroadenoma/fibroadenomatous hyperplasia	20 (50)
Atypical ductal hyperplasia and sclerosing adenosis	8 (20)
Duct dilatation	4 (10)
Intraductal papilloma	4 (10)
Fat necrosis	2 (5)
Granulomatous lobular mastitis	2 (5)
Malignant	68
Invasive ductal carcinoma	54 (79.41)
Ductal carcinoma in situ	10 (14.71)
Mucinous carcinoma	2 (2.94)
Invasive lobular carcinoma	2 (2.94)

### Interobserver agreement for all protocols

There was almost perfect interobserver agreements for all protocols; the κ value of UE, AP and FDP were 0.88, 0.92, and 0.98 (*p* < 0.001), respectively.

### Diagnostic performance

**Figure 1:**
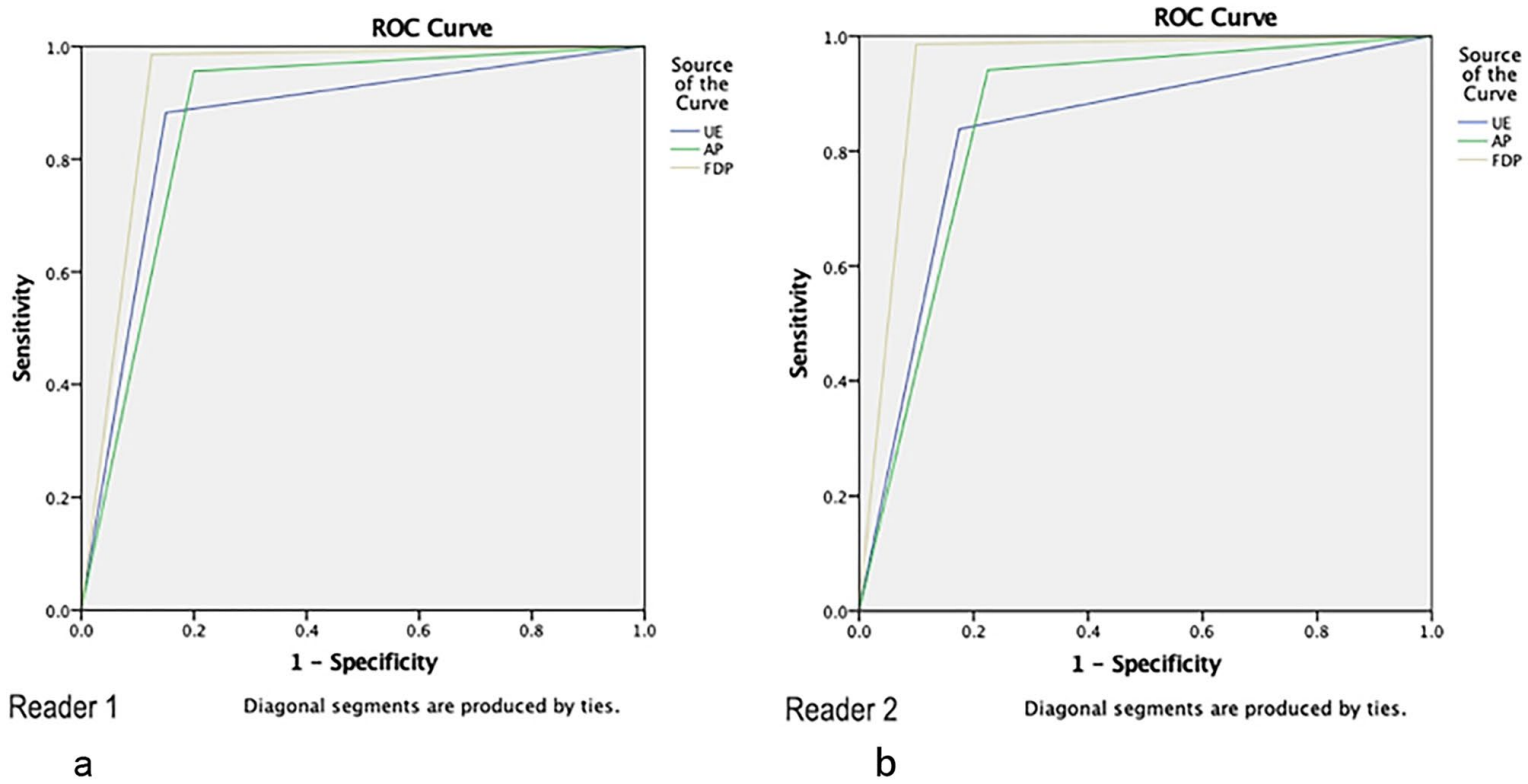
Receiver operating characteristic (ROC) curve analysis for all protocols and readers 1 (**a**) and 2 (**b**). The area under the curves (AUCs) for the unenhanced (UE), abbreviated protocol (AP), and full diagnostic protocol (FDP) for reader 1/reader 2 were 0.87/0.83, 0.88/0.86, and 0.93/0.94, respectively.

The sensitivity, specificity and AUC of the three protocols are reported in Table 2. For both readers, there was no significant difference in sensitivity between AP and FDP or between AP and UE (*p*_(*r1*)_ = 0.480, *p*_(*r1*)_ = 0.130; *p*_(*r2*)_ = 0.249, *p*_(*r2*)_ = 0.229); however, the sensitivity of UE was significantly lower than that of FDP (*p* = 0.023, *p* = 0.004), which indicated that AP was more comparable to FDP in detecting positive lesions than UE. However, the specificities of the three protocols had no significant difference (UE/AP, *p*_(*r1*)_ = 0.680, *p*_(*r2*)_ = 0.752; FDP/AP, *p*_(*r1*)_ = 0.249, *p*_(*r2*)_ = 0.074; FDP/UE, *p*_(*r1*)_ = 1, *p*_(*r2*)_ = 0.371). It should be noted that both readers found that the AUC of FDP (R1/R2:0.930/0.943) was higher than that of UE (R1/R2:0.87/0.83) or AP (R1/R2:0.878/0.858), but the difference between the latter two was not statistically significant (UE/AP, *p*_(*r1*)_ = 0.745, *p*_(*r2*)_ = 0.558; FDP/AP, *p*_(*r1*)_ = 0.026, *p*_(*r2*)_ = 0.039; FDP/UE, *p*_(*r1*)_ = 0.026, *p*_(*r2*)_ = 0.002) (Fig. 1).

**Table 2:** The sensitivity, specificity, area under the curve (AUC) and reading time for unenhanced (UE), abbreviated protocol (AP), and full diagnostic protocol (FDP).

MRI parameters	Reader	Sensitivity (%)	Specificity (%)	AUC	CI	Reading time (mean, s)
UE	Reader 1	88.24 (60/68)	85.00 (34/40)	0.866	0.788–0.944	26.58
	Reader 2	83.82 (57/68)	82.50 (33/40)	0.832	0.747–0.917	27.56
AP	Reader 1	95.59 (65/68)	80.00 (32/40)	0.878	0.799–0.957	34.08
	Reader 2	94.12 (64/68)	77.50 (31/40)	0.858	0.774–0.942	32.68
FDP	Reader 1	98.53 (67/68)	87.50 (35/40)	0.930	0.867–0.993	210.39
	Reader 2	98.5 (67/68)	90.0 (36/40)	0.943	0.885–1.000	310.13

**Figure 2:**
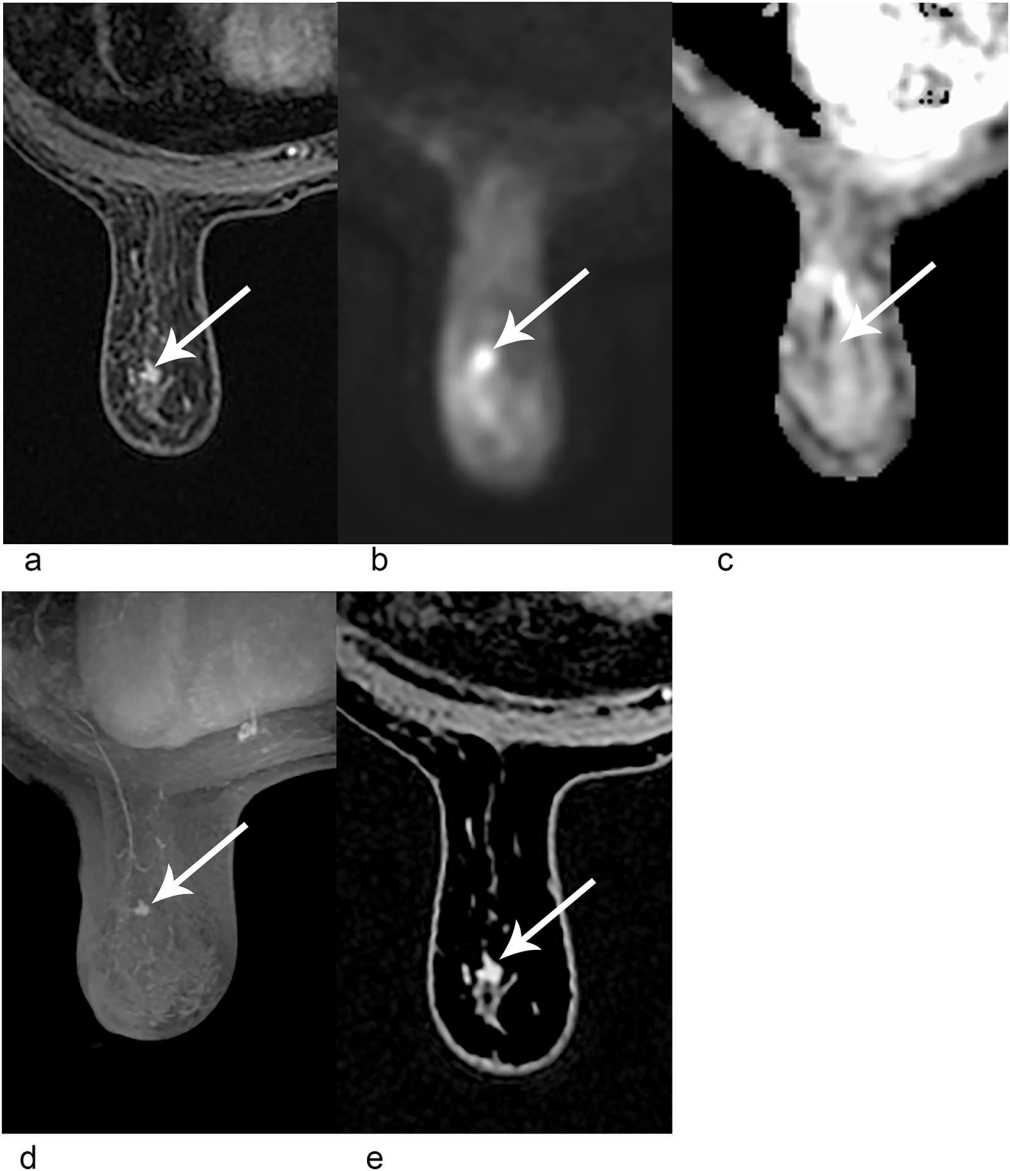
A 50-year-old female; an invasive ductal carcinoma was not found on mammography with the background of heterogeneously dense breasts. (1) Unenhanced (UE)-MRI including images of FS-T2WI (**a**), DWI (**b**), and ADC map (**c**). An irregular mass of 5 mm diameter in the left breast (arrow) was detected by FS-T2WI. In the DWI sequence, the mass presented hyperintensity with corresponding ADC value = 1.61 × 10^–3^ mm^2^/s. As the result of the ADC value was higher than the cutoff, one of the readers scored this lesion as benign, whereas the other one thought this mass was malignant, mainly due to the irregular shape. (2) Abbreviated protocol (AP)-MRI consisting of the maximum-intensity projection (MIP) (**d**) and first post-contrast subtracted (FAST) (**e**). The images showed an enhanced mass with a slightly lobulated margin on the left breast, which was classified as BI-RADS 4.

For different lesion size groups, reader 1 found that AP and UE or FDP had similar sensitivities, which was independent of size. The difference was that reader 2 found that UE had limitations in detecting lesions ≤ 10 mm, and its sensitivity was significantly lower than the other two schemes, and a representative case is shown in Fig. 2. For lesions with diameter > 10 mm, all the three protocols had high sensitivities in both readers. The sensitivities of different carcinoma sizes are shown in Table 3.

**Table 3:** Diagnostic sensitivity of unenhanced (UE), abbreviated protocol (AP) and full diagnostic protocol (FDP) in different size groups of the malignant lesions.

MRI protocols	Reader 1 (%)		Reader 2 (%)	
	≤ 10 mm	> 10 mm	≤ 10 mm	> 10 mm
UE-MRI	73.68 (14/19)	93.88 (46/49)	57.89 (11/19)	93.88 (46/49)
AP-MRI	89.47 (17/19)	97.96 (48/49)	89.47 (17/19)	95.92 (47/49)
FDP-MRI	94.74 (18/19)	100 (49/49)	94.47 (18/19)	100 (49/49)
*P*_a_ (UE vs. AP)	0.37	1	0.04	1
*P*_b_ (UE vs. FDP)	0.13	0.57	0.02	0.25
*P*_c_ (FDP vs. AP)	1	0.32	1	0.48

**Figure 3:**
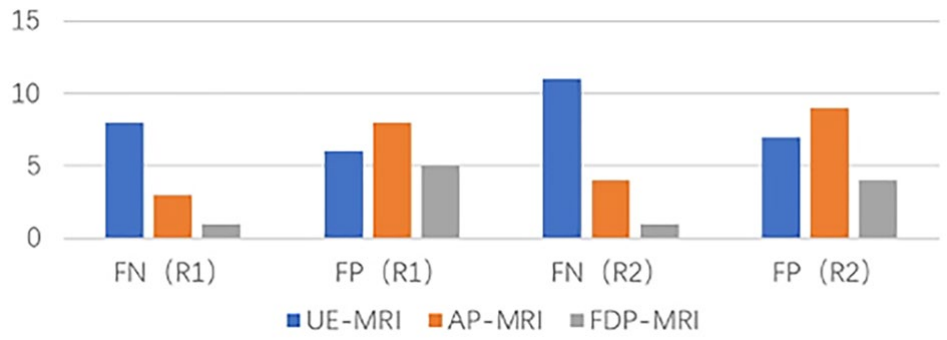
Numbers of false positive (FP) and false negative (FN) lesions using the protocols of unenhanced (UE), abbreviated protocol (AP) and full diagnostic protocol (FDP).

**Figure 4:**
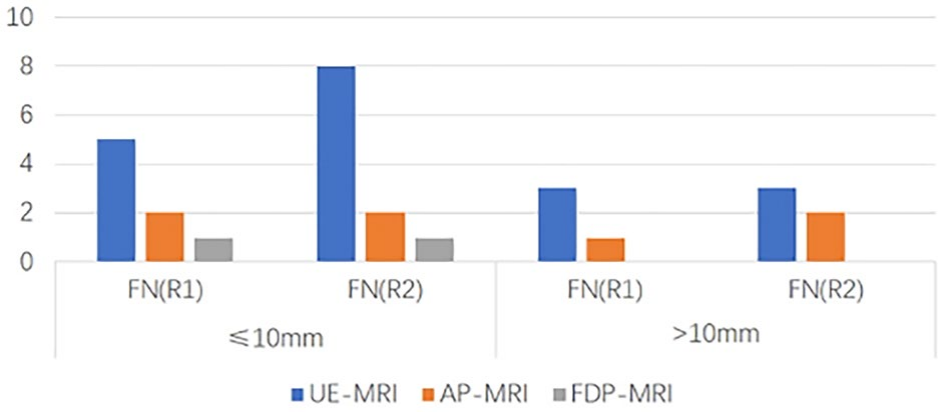
Numbers of false negative (FN) lesions using unenhanced (UE), abbreviated protocol (AP) and full diagnostic protocol (FDP) according to lesion size.

The numbers of false positive (FP) and false negative (FN) findings on each protocol is shown in Fig. 3. The numbers of FN lesions on each protocol in the subgroups of different lesion size are shown in Fig. 4. For both readers, the number of FN lesions in UE-MRI was higher than that in AP-MRI, especially for lesions with diameter ≤ 10 mm. The average diameters of the lesions detected by UE and AP were 19 mm (7–31 mm) and 16 mm (5–31 mm), respectively. Of the 8(R1)/11(R2) positive cases missed by UE, 5 (R1)/8(R2) were lesions ≤ 10 mm in diameter; the smallest tumors detected by UE were 6 mm in diameter. A total of 3(R1)/4(R2) lesions were missed by AP, of which 1(R1)/2(R2) were ≤ 10 mm in diameter and 2(R1)/2(R2) were > 10 mm in diameter; the smallest tumors detected by AP were 5 mm in diameter. For both readers, FDP detected all breast tumors > 10 mm in diameter, whereas it missed one lesion ≤ 10 mm. For FP cases, the number of misdiagnosed lesions on AP (R1 = 8; R2 = 9) was higher than that on UE (R1 = 6; R2 = 7) for both readers, whereas the number of misdiagnosed lesions was higher on UE than on FDP (R1 = 5; R2 = 4).

### Timing

In this study, the image acquisition times of UE, AP, and FDP were 5 m 8 s, 3 min, and 17 min, respectively.

The overall mean reading times of UE and AP were 27.1 s (12–45.9 s) and 33.4 s (17.8–53.9 s), respectively, whereas the average reading time of FDP was 260.3 s (127–542 s). The reading time of FDP was significantly longer than those of UE and AP (*p* < 0.001), and the shortest use time was UE (*p* < 0.001), Table 2.

## Discussion

We analyzed mammographically undetected lesions in dense breast in this study; therefore, the tumors would have been missed in the absence of supplementary examinations. With increasing scholarly research on AP and UE-MRI, there is new hope that breast MRI can be used as a rapid and economical supplementary screening scheme for dense breasts^9,16^.

Different research groups have reported consistent results^17–19^, suggesting that the sensitivity of AP ranged from 89 to 96%, and was equivalent to that of FDP. Previous reports^9,20^ indicated that the specificity of common abbreviated protocols did not differ from that of FDP. However, Grimm et al.^10^ and Chen et al.^11^ reported that the specificity of AP (52%, 86.5%), was lower than that of FDP. These results may indicate that although AP had high sensitivity similar to FDP for breast cancer, it cannot completely replace FDP in clinical practice. In our study, we further confirmed this by showing that AP had a lower AUC than FDP. In previous studies, the protocols for AP-MRI have not been consistent. Heacock et al.^21^ included T2-weighted sequences within their abbreviated protocols. However, they found that T2-weighted sequences did not improve the detection of cancer in readers. In terms of the dynamic features of pharmacokinetic, Grimm et al.^10^ showed that adding the second post-contrast sequence to AP would not affect the sensitivity or specificity of diagnosing breast cancer. Chen et al.^19^ found that the combination of common AP and DWI sequence improved the specificity from 86.5 to 95.0%. Oldrini et al.^22^ combined ultrafast MRI with the AP protocol, and the specificity of diagnosis was significantly improved, but the sensitivity did not change significantly. Therefore, further research is needed to achieve consistent standardized AP protocols.

Unlike DCE-MRI and AP-MRI, which rely on an injection of gadolinium-based contrast agent to show tissue hemodynamics, diffusion-weighted imaging measures endogenous water movement in tissues. Zhang et al.^23^ reported that compared with DCE-MRI, DWI has a higher specificity (75.6% vs 71.1%) but lower sensitivity (86.0% vs 93.2%). DWI together with apparent diffusion coefficient (ADC) maps can be used to help to distinguish malignant and benign breast lesions^23^. The previous studies found that comparisons of DWI sequences alone, integrated DWI with T2WI can improved the detection of the location of lesions, and also prevented missing lesions on DWI^24–26^. So, our study used the combination sequence as UE-MRI. Amornsiripanitch et al.^13^ concluded in a study that the average diagnostic sensitivity of UE was 72%, which was lower than the specificity (90%). They attributed the false-negative findings of UE to some special pathological types, which may exhibit higher ADC measurements due to less diffusion impedance or higher water content, such as DCIS, mucinous carcinoma or triple-negative carcinoma with extensive necrosis^13^. In addition, Pinker et al.^27^ and Rotili et al.^28^ suggested that another factor resulting in the decrease of sensitivity is the smaller tumors, especially lesions ≤ 10 mm, which are not well detected in UE-MRI blind reading studies. The typical DWI in-plane resolution (2 × 2 mm^2^) and slice thickness (3–5 mm) may bring about significant partial volume effect for small lesions, thus increasing false negative findings^29^. In our study, one of the readers found that UE had limitations in detecting lesions ≤ 10 mm. However, for lesions > 10 mm, it showed extremely high sensitivity at 93.88% for both readers, similar to previous studies^28^.

The diagnostic efficacies of AP and UE-MRI were compared for occult lesions of mammography. The common advantage of the two was that they significantly reduced the scanning time and interpretation time compared with FDP-MRI. AP is superior to UE in its sensitivity for the diagnosis of breast cancer of different sizes. Telegrafo et al.^25^ and Kang et al.^30^ used advanced read-out segmented echo planar imaging (EPI), DW, MRI, and background suppression techniques in their research, resulting in high sensitivity (93%, 94%). If the problem of low sensitivity in small size lesions could be solved, UE-MRI may be a potential supplementary screening tool for breast cancer because it does not require a gadolinium-based contrast agent, and the public is increasingly concerned about the unknown health problems of gadolinium deposition in the brain and other tissues by injecting a gadolinium contrast agent repeatedly^31^. Therefore, it is difficult to balance the benefits of AP-MRI with the issue of the safety of gadolinium deposition^32^.

The limitations of this study were as follows: First, because this study lacked a sufficient number of non-mass enhanced (NME) lesions, a subgroup analysis of mass and non-mass enhanced lesions was not performed. Avendano et al.^33^ reported that 31% of the lesions with NME on DCE-MRI could not be assessed by DWI. Heacock et al.^21^ found that AP-MRI protocol may be more challenging to detect lesions presenting as NME. Second, in the past few years, there was no unified standard for slice thickness in DWI scanning; the slice thickness used in different studies was 3–5 mm^15,25,28,34,35^. Before 2019, the “European Society of Breast Radiology” established a minimum slice thickness of 4 mm^36^. However, patients from 2015 to 2018 were included in our study, and a slice thickness of 5 mm was selected, which was based on previous studies. Therefore, this may affect the sensitivity of UE-MRI, especially for small-size lesions. In the future, we will reduce the slice thickness of DWI. Third, there are no unified guides for UE-MRI and AP-MRI interpretation. In UE-MRI, the optimal cutoff value of ADC has not been established. The b value and cutoff value of ADC based on each scholar's research are different, which may lead to differences between our study and other research results. The sequence of the AP-MRI protocol also needs to be optimized in further prospective research. Forth, in our study, patients who had been received neoadjuvant chemotherapy was excluded. In future studies, we will further compare and analyze the differences between UE and AP in evaluating the efficacy of neoadjuvant chemotherapy for breast cancer.

In conclusion, most occult lesions in mammography can be found by UE-MRI or AP-MRI, though the AUC of both UE and AP were lower than that of FDP in our study. Regarding the sensitivity of diagnosis, AP-MRI may be superior to UE-MRI, especially in lesion sizes ≤ 10 mm. However, the safety based on gadolinium use cannot be ignored in future research.

## Materials and methods

### Participants

This retrospective study was permitted by the Ethics Committee of the Second Hospital of Shandong University, approval number was KYLL-2021(LW)042, and the informed consent was obtained from all participants. The research scheme was conducted in accordance with the standards set out in the Declaration of Helsinki. From September 2015 to December 2018, 1029 consecutive women underwent breast MRI for assessment of suspicious findings on screening ultrasound or mammography, detection of additional lesions, annual screening of women with high risk factors of breast cancer, and evaluation of response to neoadjuvant chemotherapy. The criteria for excluding 555 patients were as follows: 63 patients did not undergo mammography, 216 women with breasts consisting of scattered fibroglandular tissue and almost entirely fat observed by mammography, 183 women who had been received neoadjuvant chemotherapy, 74 women without pathological results or without follow-up after MR examinations or followed-up for less than 2 years, and 19 women who had MRI findings that could not match biopsy results. Then, the mammographic images of the remaining 474 patients with dense breast tissue were interpreted by a senior radiologist of breast diseases. A total of 372 women were excluded from this study owing to BI-RADS categories 4 and 5. Finally, 102 women with negative or benign findings on mammography were considered eligible for the study. All women with MRI positive or MRI negative but suspicious lesions found by ultrasound examination underwent ultrasound-guided biopsy or surgical resection biopsy.

### MRI examination

All patients underwent MRI examinations with a 3.0-T magnet (Discovery 750, General Electric Healthcare, USA). They were imaged in the prone position, with an 8-channel dedicated breast coil.

The breast MRI protocol included axial fast spin echo (FSE) T2WI with a fat suppression sequence (repetition time [TR]/echo time [TE]: 5118/85.0, slice thickness: 5 mm, spacing: 1.0 mm), axial TI-FSE sequence (TR/TE: 484/15.6, slice thickness: 5 mm, spacing: 1.0 mm), axial single shot fat suppressed echoplanar diffusion-weighted sequence (TR/TE: 3000/49.5, slice thickness: 5 mm, spacing: 1.0 mm), and the b-values including 0 and 800 s/mm^2^, and axial T1-weighted 3D-dynamic gradient echo fat sequence (VIBRANT) (TR/TE = 3.9/1.7, slice thickness: 1.8 mm, flip angle: 5°). The dynamic contrast-enhanced images were acquired immediately after the saline and gadodiamide (rate of 2 ml/s, 0.2 mmol/kg body weight) injections. There were seven sequences with no time interval, each of which was 60 s. The total acquisition time of the full diagnostic protocol MR was 17 min.

MRI images were transferred to GE workplace software (Advantage Windows Workstation, 4.6; GE Healthcare. http://www.gehealthcare.cn); apparent diffusion coefficient, subtraction, and MIP images were automatically obtained.

### Image interpretation

The images were interpreted by two breast radiologists with 9 and 20 years of experience in breast MRI. The two readers were blinded to the medical history of all the patients, the reason for seek medical advice, previous imaging information, and the risk levels of patients were included.

Each reader independently assessed the MRI images of 102 women in three steps in random order on the Picture Archiving and Communication system (PACS) workstation. First, the images from UE-MRI were interpreted as following: the identification, localization and preliminary evaluation of lesions were on the basis of the images of T2WI and DWI. We assessed the morphological characteristics and the signal intensities of lesions on T2WI and used them as diagnostic criteria. The margins of lesions found on T2WI were divided into regular and irregular margins. In addition, on the T2-weighted images, the signal intensities of hyperintense, isointense, or hypointense were based on fibrous gland tissue as the contrast standard. Then, we detected the suspicious hyperintense signal lesions in DWI sequence, in which we selected the region of interest (ROI), and measured its apparent diffusion coefficient (ADC) value (based on the method of “single ROI”)^37^. According to previous data, the cutoff of ADC value for differentiating benign and malignant breast lesions was 1.18 × 10^–3^ mm^2^/s^38^. The diagnostic criteria were as follows: ADC value > 1.18 × 10^–3^ mm^2^/s, hypointense signal of a round or oval shape, circumscribed margin masses indicated benign features; ADC value less than the cutoff, high signal intensity of an irregular shape, not circumscribed (irregular or spiculated) margin masses were considered malignant lesions. Readers measured the time required for diagnosis and recorded the results. Second, AP images (FAST and MIP) were read. FDP images were the last to be interpreted. For the last two procedures, the time was calculated, respectively; the results were recorded on the basis of the Breast Imaging-Reporting and Data System (BI-RADS). To minimize the impact of memory, the interval between interpretations for all cases was greater than > 15 days.

### Statistical analysis

All MRI findings were evaluated based on the American College of Radiology (ACR) BI-RADS Atlas Fifth Edition, BI-RADS 1–3 was considered negative and BI-RADS 4–5 was positive. The Chi-square test was performed to calculate the area under the curve (AUC), specificity, sensitivity, and 95% confidence interval (CI) of UE, AP, and FDP. The size of the breast malignant tumor was assessed by pathology. Tumors were divided into two subgroups (≤ 10 mm and > 10 mm in diameter); the sensitivity of each MRI parameter was also separately assessed. The paired *t*-test was used to evaluate the reading time for all protocols; κ-scores were used for assessing the inter-reader agreement on the different protocols.

SPSS statistical software, version 23.0 (IBM, Armonk, New York, USA. http://www.ibm.com) was performed for all statistical analyses; *p-*values less than 0.05 were considered to be statistically significant.
